# Resveratrol, Piceatannol, Curcumin, and Quercetin as Therapeutic Targets in Gastric Cancer—Mechanisms and Clinical Implications for Natural Products

**DOI:** 10.3390/molecules30010003

**Published:** 2024-12-24

**Authors:** Paulina Warias, Paulina Plewa, Agata Poniewierska-Baran

**Affiliations:** 1 Doctoral School, University of Szczecin, Mickiewicza 18, 70-384 Szczecin, Poland; paulina.warias@phd.usz.edu.pl; 2 Department of Physiology, Pomeranian Medical University in Szczecin, Powstancow Wielkopolskich 72, 70-111 Szczecin, Poland; paulina.plewa@op.pl; 3 Institute of Biology, University of Szczecin, Felczaka 3c, 71-412 Szczecin, Poland

**Keywords:** natural products, gastric cancer, resveratrol, piceatannol, curcumin, quercetin

## Abstract

Gastric cancer remains a significant global health challenge, driving the need for innovative therapeutic approaches. Natural polyphenolic compounds such as resveratrol, piceatannol, curcumin, and quercetin currently show promising results in the prevention and treatment of various cancers, due to their diverse biological activities. This review presents the effects of natural compounds on important processes related to cancer, such as apoptosis, proliferation, migration, invasion, angiogenesis, and autophagy. Resveratrol, naturally found in red grapes, has been shown to induce apoptosis and inhibit the proliferation, migration, and invasion of gastric cancer cells. Piceatannol, a metabolite of resveratrol, shares similar anticancer properties, particularly in modulating autophagy. Curcumin, derived from turmeric, is known for its anti-inflammatory and antioxidant properties, and its ability to inhibit tumor growth and metastasis. Quercetin, a flavonoid found in various fruits and vegetables, induces cell cycle arrest and apoptosis while enhancing the efficacy of conventional therapies. Despite their potential, challenges such as low bioavailability limit their clinical application, necessitating further research into novel delivery systems. Collectively, these compounds represent a promising avenue for enhancing gastric cancer treatment and improving patient outcomes through their multifaceted biological effects.

## 1. Introduction

Gastric cancer (GC) remains one of the leading causes of cancer-related mortality worldwide, necessitating the exploration of novel and effective therapeutic strategies. It has been proven that regular daily consumption of fruits and vegetables protects against diseases and the development of cancers, which has been confirmed by the results of case-control studies. In recent years, naturally occurring polyphenolic compounds have garnered significant attention due to their diverse biological activities and potential roles in cancer prevention and treatment [1]. This text delves into the therapeutic potential of four such compounds—resveratrol, piceatannol, curcumin, and quercetin—highlighting their sources, biological effects, mechanisms of action, and implications in the management of gastric cancer and associated inflammatory processes.

The significant influence of resveratrol (RSV) and other polyphenolic natural compounds on GC has already been described in our review about sirtuins [2]—the seven enzyme proteins that are activated by RSV, as well as their expression, affect different stages of GC development. In this paper, we wanted to clearly indicate how RSV affects other molecular therapeutic targets in GC, and how other natural substances, like piceatannol, curcumin, and quercetin (often valued in folk medicine, or as an alternative method of supporting therapy), can significantly affect GC cells and the course of this disease.

As we will point out in this review, dietary supplementation (or another way of administration) of resveratrol, piceatannol, curcumin, and quercetin can effectively eliminate the progression of GC cells.

Resveratrol, a well-studied stilbene found predominantly in red grapes and wine [3], exhibits a broad spectrum of biological activities, including antioxidant, anti-inflammatory, and anticancer effects [4]. Studies have demonstrated its ability to inhibit proliferation, induce apoptosis, and suppress the migration and invasion of gastric cancer cells through various molecular pathways such as nuclear factor kappa B (NF-κB) [5] and Wnt/β-catenin [6] signaling. Moreover, resveratrol has shown promise in enhancing the efficacy of conventional chemotherapeutic agents and overcoming multidrug resistance, although challenges related to its bioavailability persist [7].

Piceatannol, a metabolite of resveratrol present in foods like grapes, white tea, and passion fruit [4], shares similar chemopreventive properties. Research indicates that piceatannol can inhibit proliferation and induce autophagy in gastric cancer cells by modulating signaling pathways and interacting with key proteins such as beclin-1 [8]. Its potential synergistic effects with other anticancer agents underscore its relevance in comprehensive cancer therapy approaches.

Curcumin is the main secondary metabolite of *Curcuma longa* and other *Curcuma* spp. Curcumin is commonly used as a coloring agent as well as a food additive; curcumin has also shown some therapeutic activities [9]. Curcumin is considered an antioxidant due to the β-diketone group in its structure. The most important mechanisms by which curcumin is able to promote the majority of its activities are by the inhibition of superoxide radicals, hydrogen peroxide, and the nitric oxide radical. In addition, curcumin is also able to increase the activity of xenobiotic detoxifying enzymes both in the liver and kidneys, protecting against carcinogenesis processes [10]. It has a variety of anti-inflammatory properties, and in addition to its potent antioxidant activity, its mechanisms of action include the inhibition of several cell signaling pathways at multiple levels, effects on cellular enzymes such as cyclooxygenase and glutathione S-transferases, immunomodulation, and effects on angiogenesis and cell adhesion. Curcumin’s ability to affect gene transcription and induce apoptosis in preclinical models may be of particular relevance to cancer chemoprevention and chemotherapy in patients. Although the low systemic bioavailability of curcumin after oral administration may limit the availability of sufficient concentrations for pharmacological action in some tissues, the attainment of biologically active levels in the gastrointestinal tract has been demonstrated in animals and humans. There are now sufficient data to support a Phase II clinical evaluation of oral curcumin in patients with invasive malignancies or pre-invasive lesions of the gastrointestinal tract, particularly the colon and rectum [11].

Quercetin is classified as a flavonol, one of six subclasses of flavonoid compounds. By definition, quercetin is an aglycone, with no sugar attached. It has a brilliant lemon-yellow color and is fairly soluble in alcohol but poorly soluble in water [12]. It is ubiquitous in foods, including vegetables, fruits, tea and wine, as well as in countless dietary supplements, and is claimed to have beneficial effects on health. This includes protection against various diseases such as osteoporosis, some forms of cancer, lung and cardiovascular disease, but also against aging. In particular, it has been suggested that quercetin’s ability to capture highly reactive species, such as peroxynitrite and hydroxyl radical, is involved in these possible beneficial health effects [13]. Although metabolic conversion attenuates its biological effects, active aglycone can be generated from glucuronide conjugates by increased β-glucuronidase activity during inflammation. Regarding its association with molecular targets relevant to cancer prevention, quercetin aglycone has been shown to interact with certain receptors, in particular the aryl hydrocarbon receptor, which is involved in the development of cancers induced by certain chemicals. Quercetin aglycone has also been shown to modulate several signal transduction pathways involving MEK/ERK and Nrf2/keap1, which are involved in inflammation and carcinogenesis processes. Studies have shown that the dietary administration of this flavonol prevents chemically induced carcinogenesis, particularly in the colon, while epidemiological studies have shown that quercetin intake may be associated with the prevention of lung cancer. Dietary quercetin is therefore a promising agent in cancer prevention [14].

Collectively, these natural compounds offer promising avenues for the prevention and treatment of gastric cancer through their multifaceted biological effects and low toxicity profiles. This compilation underscores the importance of continued research into their mechanisms of action, optimization of bioavailability, and integration into existing cancer treatment paradigms to improve patient outcomes and combat this prevalent disease effectively.

## 2. Resveratrol

**Figure 1:**
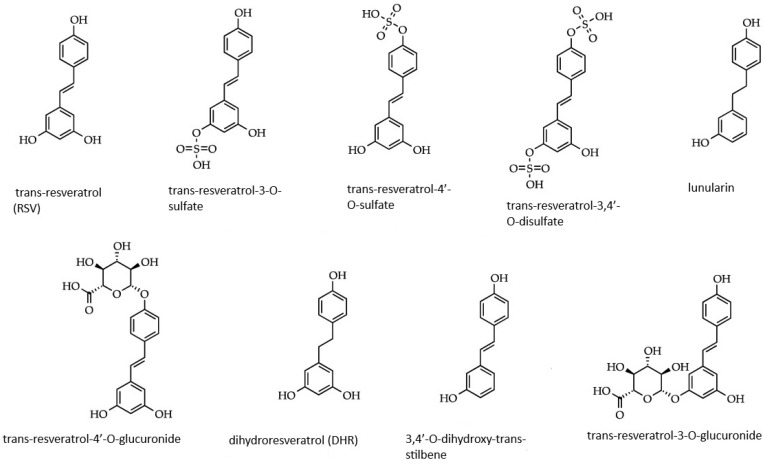
Forms of resveratrol metabolites.

Resveratrol (trans-3,4,5-trihydroxystilbene) is an increasingly studied substance found naturally in red wine [3], and in many varieties of red grapes [15]. It is also found in rhubarb [16], fruits such as blueberries [17], and peanuts [18]. Resveratrol was first described and isolated from the white hellebore (*Veratrum grandiflorum*) by Japanese researcher Takaoka in 1939 [19]. Resveratrol comes in several forms, as shown in Figure 1. It has two phenolic rings, monophenol and diphenol, which are linked to each other by a styrene double bond, and occurs in both cis and trans isomeric forms. Trans-resveratrol appears to be the more common and stable natural form [20]. The molecule described has three hydroxyl groups, which are involved in free radical scavenging and metal chelation. The presence of hydroxyl groups also facilitates interaction with macromolecules [21]. Resveratrol can exhibit antioxidant, anti-inflammatory, anticancer, antimicrobial, anti-neurodegenerative, estrogenic, and anti-estrogenic properties [4].

### Resveratrol in Gastric Cancer and Inflammation

A study was conducted using the HGC-27 and AGS gastric adenocarcinoma cell lines in which the effects of resveratrol (RSV, Res) on parameters such as migration, invasion, proliferation, and apoptosis were examined. The tests performed showed that proliferation was dramatically inhibited in both cell lines compared to controls. The apoptosis assays showed a significant increase in the apoptotic cells of both cell lines, after RSV treatment. In an experiment showing the effect of resveratrol on the migration and invasion of HGC-27 and AGS cell lines, a significant inhibition of both properties was detected. To further confirm the effect of resveratrol on gastric cancer, a mouse model of GC (with implanted HGC-27 and AGS cells) was created. Analysis of the tumor size showed a reduction in tumor size after RSV treatment, and inflammatory cell infiltration was significantly alleviated after RSV treatment [22].

There was also a study that showed how appropriate concentrations of resveratrol are important in gastric cancer therapy. The experiment was also performed on human gastric adenocarcinoma cell lines AGS and MKN45. The cancer cells showed significant sensitivity to treatment with RSV, which inhibited viability in a dose-dependent (25–200 μM) manner in both cell lines. In the second experiment, lower concentrations of resveratrol (5–12.5 µM) showed no statistically significant growth inhibition. The effect of RSV on the invasion potential of gastric cancer cells was determined, and the results showed that it reduced the invasion potential in both AGS and MKN45 cell lines in a dose-dependent manner. It was observed that treatment with a high dose of resveratrol (5–25 μM) significantly reduced the invasion potential of AGS cells from 60 to almost 90% compared to control conditions. Meanwhile, RSV significantly reduced MKN45 invasion from 75 to almost 95% compared to the control group. Interestingly, RSV 25 µM significantly reduced invasion (90 and 95% in AGS and MKN45, respectively) compared to similar concentrations, with less effect on cell viability (45 and 40% in AGS and MKN45, respectively). Based on these results, it can be concluded that RSV may affect various processes involved in carcinogenesis, depending on the concentration used. Potential mechanisms of cell invasion that were inhibited were also included in the study. For this purpose, superoxide dismutase (SOD) and NF-κB were examined. The results showed that cells treated with RSV led to an increase in SOD activity in a dose-dependent manner compared to the control conditions in both cell lines; however, this increase was statistically significant only when gastric cancer cells were treated with the highest concentration of the substance. In a study showing that RSV decreases NF-κB transcriptional activity in an experimental model, they analyzed changes in heparanase activity, which was linked to NF-κB expression in gastric cancer cells after RSV treatment. Heparanase activity was reduced in AGS cells treated with 5–25 µM RSV, so it can be concluded that resveratrol reduces heparanase activity in gastric cancer cells, which correlates with increased SOD activity and the inhibition of NF-κB transcriptional activity [5]. The destructive effect of RSV on adenocarcinoma cells is of great clinical significance, because gastric adenocarcinoma is the most common type of gastric malignant cancer, that makes up 90–97% of all malignant gastric tumors.

Another study on the effect of resveratrol on gastric cancer concerned the regulation of microRNA, specifically miR-155-5p, whose overexpression leads to oncogenesis. Cancer cell lines SGC7901, GES-1, MGC803, and AGS were used for the study. The results revealed that miR-155-5p expression decreased with RSV treatment in a dose-dependent manner. The researchers also showed the inhibition of proliferation, invasion, and migration, as well as morphological changes in the cells. Additional studies conducted on the SGC7901 line showed that RSV significantly decreased the expression of claudin-1, c-Myc, cyclin D1, and Bcl-2, and increased the expression of caspase-3 [23]. Studies confirm the effect of RSV on the induction of apoptosis in SGC7901 cells [24].

The next goal in the study of RSV’s therapeutic properties was to determine its effects on the Wnt/β-catenin signaling pathway. MGC-803 gastric cancer lines were used in this study, and the results confirmed that Res reduced the expression of the three important components of the Wnt signaling pathway—β-catenin, c-Myc, and cyclin D1—at the mRNA and protein levels. The effect of resveratrol was also confirmed; it significantly inhibited MGC-803 cell proliferation in a dose-dependent manner and induced apoptotic morphological changes in MGC-803 cells [6]. To observe the broad spectrum of RSV effects, further studies were undertaken. For this purpose, the effect of the substance on MALAT1 (transcript 1 of metastasis-associated lung adenocarcinoma), whose expression is increased in gastric cancer, was investigated. An experiment on the BGC823 cell line confirmed that Res inhibited the migration and invasion of human gastric cancer cells by inhibiting MALAT1-mediated epithelial-to-mesenchymal transition. This phenomenon was influenced by the downregulation of MALAT1 lncRNA expression by resveratrol [24]. Referring to MALAT1, other studies show that RSV inhibits proliferation and the migration and invasion of human cells in gastric cancer [25], e.g. through the MALAT1/miR-383-5p/DDIT4 signaling pathway in the SGC7901 cell line [26].

A very important aspect in the treatment of gastric cancer is the development of multidrug resistance (MDR) by these cells. Experiments were conducted on human gastric cancer cell lines EPG85-257RDB (RDB)—resistant to daunorubicin and EPG85-257RNOV (RNOV)—resistant to mitoxantrone, in addition to EPG85-257P (control), which is sensitive to cytostatic drugs. The aim of the study was to demonstrate the effect of 3,5,4′-trihydroxy-trans-stilbene (RSV) on the expression of the ATP-binding cassette genes of subfamily B member 1, annexin A1 (ANXA1), and thioredoxin (TXN), as well as the proteins encoded by these genes, which are associated with MDR. The results indicate that resveratrol can reduce cancer cell resistance by affecting the downregulation of the expression of a number of MDR-related genes and proteins [6]. Studies were also conducted in which Res had an adjuvant role where its positive role was proven. The results showed that treatment together with cisplatin (DDP) significantly affected the inhibition of cell viability, promoted cell apoptosis, and induced G2/M phase arrest in AGS cells. In addition, Res in combination with DDP was found to significantly increase the levels of Bax, cleaved poly-ADP-ribose polymerase (PARP), glucose-regulated protein 78 (GRP78), PRKR-like ER kinase (PERK), eukaryotic translation initiation factor 2α (p-eIF2α), CCAAT/homologous to enhancer-binding protein (CHOP), and cleaved caspase-12, while Bcl-2 expression was downregulated after simultaneous RES/DDP treatment. In addition, simultaneous RES/DDP treatment significantly increased the levels of phosphorylated cyclin-dependent kinase 1 (p-CDK1, Tyr15), p21Waf1/Cip1, and p27Kip1, and decreased the protein levels of Cdc25C. These results indicate that RSV is a promising adjuvant for DDP during gastric cancer chemotherapy [27]. The effect of resveratrol on selected GC carcinogenesis processes is presented below in Table 1.

Another study conducted on the AGS cell line confirmed the ability of RSV as an adjuvant in chemotherapy along with cisplatin. The results showed that RSV/DDP increased ß-galactosidase activity, ROS levels as a key marker of oxidative stress, the expression of p53, p38, p16, p21, and MMP-2 genes, and induced G0/G1 cell cycle arrest. In addition, telomerase activity, the expression of pro-inflammatory genes, and cell invasion were suppressed [28]. Further evidence of the positive effect of RSV as an adjuvant is its effect on the alteration of the daunorubicin resistance of the SGC7901/DOX (daunorubicin-resistant) cell line. Moreover, the substance has the ability to reverse resistance and prevent cell migration by suppressing EMT (epithelial mesenchymal transition) in gastric cancer through the modulation of the PTEN/Akt signaling pathway. Resveratrol synergizes with DOX in inhibiting tumor growth [31].

Studies confirm that resveratrol enhances the effects of chemotherapeutics such as 5-fluorouracil [32] and cisplatin [28]. In an in vivo study of resveratrol’s efficacy, it was described to be combined with zeolite imidazole frameworks (ZIFs), which are an important subclass of metal–organic frameworks (MOFs). The HGC-27 tumor xenograft model was used, and mice were divided into groups in which Res and Res@ZIF-90 were injected via the tail vein. As a result, the group that was injected with Res@ZIF-90 showed a better ability to inhibit tumor growth compared to the Res group. This result was attributed to the fact that ZIF-90 improved the delivery efficiency and tumor enrichment capacity of Res in vivo [33]. To evaluate the effect of RES on the prometastatic properties of GC-MSCs in vivo, HGC cells were used to create models of peritoneal metastasis in nude mice. The results suggest that GC-MSCs are a promising target for Res in inhibiting GC metastasis, and that Wnt/β-catenin signaling plays an important role in this process [30].

Resveratrol has also a frequently described anti-inflammatory effect on the body, and it is one of the most important preventive measures taken against cancer. The inflammatory process promotes a microenvironment conducive to the development of cancers, and as a result, many of them develop in places where chronic inflammation or tissue regeneration occurs. Resveratrol is absorbed in large quantities by enterocytes after oral administration [34], but only a small portion of this compound ingested with the diet reaches the bloodstream and body tissues [35]. In addition, due to its complex structure and high molecular weight, the well-functioning metabolism of resveratrol in the liver and intestine leads to a bioavailability of trans-resveratrol after an oral administration of about 12% [36]. The inflammatory response is a complex, multilevel process involving a wide variety of cell types, consisting of the triggering system, sensory mechanism, signal transmission, and production of inflammatory mediators. Inflammation is an adaptive response that can be triggered by various danger signals [37], which include invasion by microorganisms (bacteria, fungi, viruses, protozoa), parasites, or tissue damage (by toxins and venom, among others) [38]. This is a very important mechanism in our body because pathogens as well as toxins lead to cancer. Exogenous and endogenous signaling molecules are known as pathogen-associated molecular patterns (PAMPs) and damage-associated molecular patterns (DAMPs), respectively [39]. Both PAMPs and DAMPs are recognized by various pattern recognition receptors (PRRs), such as Toll-like receptors (TLRs) [40]. PRR activation induces intracellular signaling cascades such as kinases and transcription factors [41]. The aforementioned signaling pathways can promote the production of various inflammatory mediators (such as cytokines) for the development of inflammation [42].

In studies on the anti-inflammatory effects of resveratrol, it was shown to inhibit the splenic cell proliferation induced by concanavalin A (ConA), interleukin (IL)-2, or allo-antigens, and more effectively prevent lymphocytes from producing IL-2 and interferon-gamma (IFN-γ), and macrophages from producing tumor necrosis factor alpha (TNF-α) or IL-12 [43]. Resveratrol has been found to induce a dose-dependent suppression of IL-1α, IL-6, and TNF-α production and to reduce both mRNA expression and IL-17 protein secretion in vitro [44]. Dietary resveratrol supplementation is able to improve the expression of the proteins zonula occludens-1, occludin, and claudin-1 to reduce intestinal permeability in vivo [45]. Resveratrol treatment also reduced the expression of inflammatory factors, the glycation end product receptor (RAGE), NF-kB (P65), and nicotinamide adenine dinucleotide phosphate (NADPH) oxidase 4 (NOX4), and improved the pathological structure of the kidney [46]. Moreover, as a natural precursor of resveratrol, polydatin (a resveratrol glycoside isolated from Polygonum cuspidatum) significantly reduced the expression of IL-6, IL-1β, and TNF-α induced by Mycoplasma gallisepticum both in vivo and in vitro, suggesting that polydatin also has anti-inflammatory effects [47].

The anti-inflammatory effect of this compound was also demonstrated in a rat model of carrageenin-induced paw edema [48]. In addition, it has been reported that resveratrol preconditioning modulates the inflammatory response of the hippocampus after global cerebral ischemia in rats [49]. It has also been documented that resveratrol is able to suppress nerve inflammation mediated by microglia, protect neurons from inflammatory damage, and alleviate airway inflammation caused by asthma and airway remodeling [50]. Heat stress can induce the production of reactive oxygen species (ROS), disrupt the antioxidant system, and cause damage to immune organs in vivo [51]. A report was published in which a research group noted that resveratrol supplementation in broiler diets was effective in partially mitigating the deleterious effects of heat stress on intestinal barrier function by restoring damaged villi and crypt structures, altering the expression of the intestinal heat-shock protein mRNA, secreting immunoglobulin A and close junction-related genes, and inhibiting pro-inflammatory secretion [52]. It has been found that the dietary supplementation of broilers with resveratrol can partially reverse the adverse effects of heat stress on immune organ growth by restoring the redox state and inhibiting apoptosis [53]. Importantly, a later study showed that resveratrol can attenuate the innate immunity and inflammatory response induced by heat stress by inhibiting the activation of PRR signaling in the spleen of broilers [54].

On the other hand, resveratrol may exert anti-inflammatory properties by inhibiting the production of ROS and nitric oxide (NO), molecules that can contribute to the tumorigenesis process. The oxidative stress caused by the accumulation of ROS plays a role in promoting inflammation in a broad spectrum of diseases, such as chronic inflammation and cancer [55]. It was found that resveratrol was able to strongly inhibit NO production in activated macrophages, as well as strongly reduce cytosolic inducible nitric oxide synthase (iNOS) and steady-state mRNA levels [56]. Dietary supplementation with resveratrol can also effectively eliminate free radicals and enhance SOD, CAT, and GPX [57]. Babu et al. [58] showed that the cytoprotective effect of resveratrol is mainly due to the mitigation of mitochondrial ROS. A recent study by Kortama et al. [59] showed that resveratrol increased the antioxidant and anti-inflammatory activity of the liver against chronic unpredictable mild stress-induced depression in an animal model, which was explained by the normalization of total antioxidant capacity, glutathione, malondialdehyde (MDA), NF-kB, TNF- α, and myeloperoxidase. In addition, resveratrol decreased iNOS mRNA expression and protein expression in LPS-stimulated intestinal cells in a dose-dependent manner, resulting in reduced NO production. Similarly, resveratrol inhibited iNOS and IL-6 expression in LPS-treated RAW264.7 cells in a dose-dependent manner, and therefore inhibited NO production and IL-6 secretion [60].

## 3. Piceatannol

**Figure 2:**
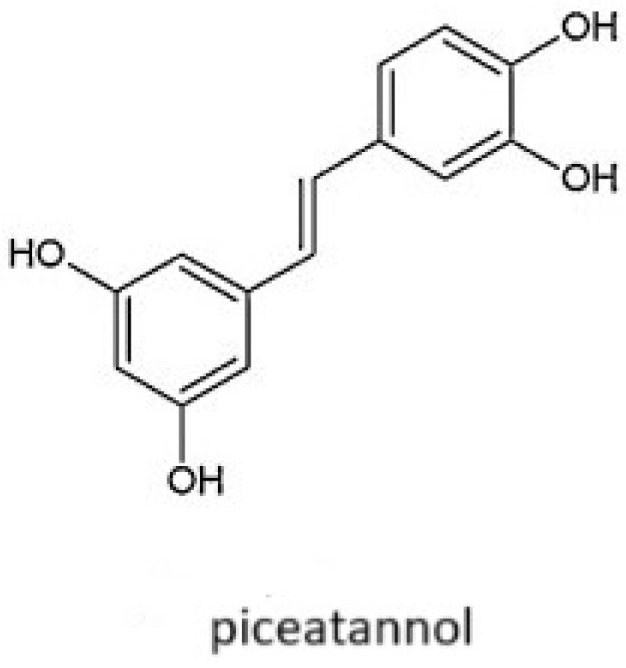
Form of piceatannol.

Piceatannol (3,3′,4,5′-trans-trihydroxystilbene) is a naturally occurring polyphenolic chemical compound that is a metabolite of resveratrol, commonly found in grapes, white tea, passion fruit [4], Asian legumes, and Korean rhubarb [61]. Form of piceatannol (presented in Figure 2) has a wide range of biological activities, such as anticancer, antioxidant, anti-inflammatory, and immune-regulating effects [62]. It exhibits various biological activities, such as skin protection against ultraviolet B (UVB) radiation [63], the inhibition of melanogenesis, the promotion of collagen synthesis [64], the vasorelaxant effect [65], and Sirt1 (sirtuin 1) induction activity [66].

### Piceatannol and Gastric Cancer

The resveratrol metabolite piceatannol is also a noteworthy component, which in studies conducted on SGC-7901 cells, induced a dose-dependent decrease in the enhancement of STAT3 phosphorylation induced by IGF-1, which is implicated in the involvement in the inhibition of proliferation, invasion, migration, and angiogenesis [67].

Piceatannol was found to effectively inhibit the proliferation of several human gastric cancer cell lines. Autophagic flow is increased by piceatannol treatment and correlates with the inhibition of cell proliferation and colony formation. In addition, the results indicate a direct interaction between piceatannol and Beclin-1, which decreases Beclin-1 phosphorylation activity. It is also noteworthy that piceatannol impairs Beclin-1 binding to Bcl-2 and enhances the recruitment of UV resistance-related gene protein binding, which further triggers Beclin-1-dependent autophagy signaling. Heterograft models confirmed that piceatannol affects tumor growth inhibition and induces strong synergistic effects with everolimus (mTOR kinase inhibitor) in vivo [8]. SGC7901 cells were injected subcutaneously into mice to create a xenograft model. The mice were divided into groups, and a combination of piceatannol (5 mg/kg^−1^) and everolimus (10 mg/kg^−1^) was administered in one group. All therapies were well tolerated, with no apparent animal weight loss. Treatment with these substances as separate chemotherapeutics had minimal effect on tumor growth, but the combination of piceatannol and everolimus significantly inhibited tumor growth. Moreover, the inhibition of tumor progression in the combination treatment group persisted for at least a week after the drug was discontinued. For the experimental endpoint, tumor tissue was harvested, and Western blot analysis showed that the levels of Beclin-1 phosphorylation at Ser-295, and mTOR at Ser-2448 were significantly reduced in the combination group, while Beclin-1 phosphorylation at Thr119 was significantly promoted. These results indicate that combination treatment with piceatannol and everolimus may synergistically increase autophagy flux through the mTOR and Beclin-1 signaling pathways. Consistent with the above results, the LC3B-I/II and Bax/Bcl-2 ratios, as well as SQSTM1/p62 degradation, were increased after combination therapy with piceatannol and everolimus. Markers of cell proliferation, cell death, and autophagy were also examined by immunohistochemical (IHC) staining. A low expression of Ki67 and PARP and high expression of Beclin-1 and LC3B were found in tumor tissues of xenografts after combination therapy. In conclusion, piceatannol in combination with everolimus can induce autophagic cell death, resulting in potent anticancer effects in vivo [8]. The effect of piceatannol on selected GC carcinogenesis processes is presented below in Table 2.

## 4. Curcumin

**Figure 3:**
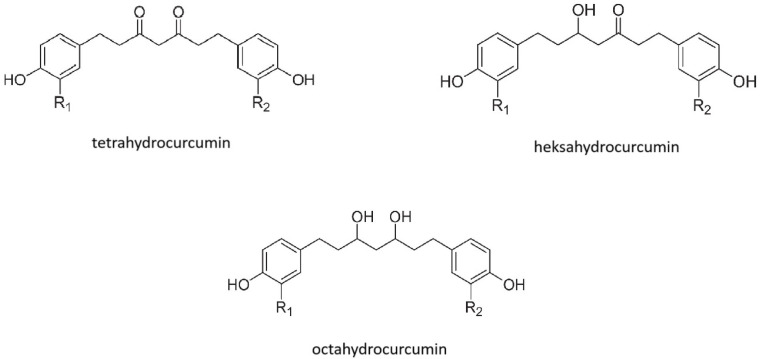
Forms of curcumin metabolites.

Curcumin is a component of the Asian spice turmeric [68]. It can be classified as a phytoextract, which is characterized by a yellow-orange pigment. Curcumin (1,7-bis(4-hydroxy-3-methoxyphenyl)-1E,6E-heptadiene-3,5-dioneor diferuloyl methane) was first isolated in 1870 from the *Curcuma longa* plant, and since then it has been widely used as a flavor complement [69]. The described molecule contains three reactive functional groups (as shown in Figure 3): a diketone group and two phenolic groups. With the help of non-covalent and covalent bonds, it can interact with many biomolecules [70]. Numerous studies have shown that curcumin has antilipidemic, hypoglycemic, anticancer, anti-inflammatory, antibacterial, antiviral, and antioxidant effects [69].

### 4.1. Curcumin and Gastric Cancer

There have been many studies on the positive use of curcumin in the treatment of GC. One study used the human gastric cancer cell line SGC-7901 and showed that curcumin reduced the migration, invasion, and proliferation of cancer cells compared to controls. Furthermore, it was observed that this molecule significantly multiplied the amount of microRNA-miR-34a, which is an antagonist of carcinogenesis in GC. This also translated into a lower expression of proteins: Bcl-2, CDK4, and cyclin D1 [71]. Another study used the HGC-37 and MKN-45 cell lines, and here it was also shown that the use of curcumin inhibited migration and invasion. In addition, in vitro apoptosis, in vivo inhibition of carcinogenesis, and manifestations of increased cell cycle arrest were observed. In the next step, the mechanism directly related to the regulation of GC progression by curcumin was analyzed. miR-194-5p is a target of many circRNAs. In this study, circ_0056618 was studied. This type of microRNA is significantly elevated in GC and affects increased proliferation and migration, and curcumin treatment inhibits circ_0056618 expression in GC cells, which translates into reduced disease progression. Interestingly, miR-194-5p, which is mainly responsible for stopping the growth of cancer cells, shows minimal expression in cells and tissues affected by GC, and circ_0056618 may negatively affect the regulation of the level of this microRNA. Increasing the levels of miR-194-5p along with the use of curcumin effectively reversed the effects of circ_0056618 [72]. In an experiment carried out again on two cell lines, SGC-7901 and BGC-823, the effect of different concentrations of curcumin on proliferation, apoptosis, and autophagy was studied. The smallest concentration of 10 μm used did not cause significant changes. A major effect was observed at 20 μm, and upon increasing the dose to 40 μm, the rate of both apoptosis and autophagy accelerated, and proliferation was significantly inhibited. Moreover, researchers were interested in tophosphatidylinositol-3 kinase (PI3K) along with proteins closely related to the p53 signaling pathway. The results indicate that curcumin has the ability to activate the p53 signaling pathway as a result of the upregulation of p53 and p21, which translates into the inhibition of the PI3K pathway as a consequence of the downregulation of PI3K, p-mTOR, and p-Akt [73]. Similar conclusions were drawn during a study on the AGS cell line, where curcumin was used in higher concentrations, i.e., 50, 75, and 100 μm, in order to determine the effect of this substance on the expression of the PI3K, Akt, and mTOR genes. The inhibitory role of curcumin increased with increasing dose. The greatest effect was observed at 100 μm, where Akt expression decreased by approximately 65% compared to the control [74]. Interesting results were obtained during a study of two human gastric cancer cells, AGS and HGC-27, during which it was discovered that curcumin significantly affects the regulation of iron, GSH (glutathione), MDA (malondialdehyde), and ACSL4 (ferroptosis marker) levels while reducing the level of lipid ROS. This may indicate the stimulating role of curcumin in the process of ferroptosis in GC cells [75]. In the case of human gastric cancer cells (hGCCs), it has been shown that 4 h of therapy with 20 μm of curcumin affects significantly increased ROS concentrations in cancer cells. In addition, a positive feedback was confirmed between the increase in curcumin concentration and ROS production in hGCC, demonstrating the pro-oxidative role of curcumin at higher concentrations. With the increase in ROS caused by curcumin, one of the mechanisms of anticancer action of this molecule is activated, associated with the reduced proliferation, migration, and initiation of apoptosis of cancer cells [76]. Another study looked at the effects of GC curcumin on angiogenesis. The study included GC-MSCs (mesenchymal stem cells derived from gastric cancer cells). The results showed that curcumin significantly reduced the expression of fibroblast proteins such as vimethine and α-SMA in GC-MSC. Such treatment also inhibited the migration and colony formation of GC-MSCc-arranged HUVECs (human umbilical vein endothelial cells). Moreover, curcumin has been observed to inhibit NF-κB signaling and produce VEGFs (vascular endothelial growth factors) [77]. During an experiment using the BGC-823 human gastric cancer cell line and male BALB/c mice, the interaction of curcumin with current chemotherapy using 5-FU (5-fluorouracil) and oxalipatine was checked. The interaction of the factors contributed to the downregulation of the Bcl-2 protein and mRNA expression. In addition, there was an upward regulation of the expression of Bax and caspases 3, 8, and 9. Moreover, the use of curcumin significantly reduced the growth of BGC-823 xenograft tumors. This may indicate the potential of curcumin in cooperation in many types of chemotherapy [78]. Another study also used male BALB/c mice that had AGS gastric cancer cells inserted subcutaneously (1 mg/kg daily). The results showed that curcumin was significantly reduced in tumor reduction. The antiproliferative and pro-apoptotic activity of curcumin has been confirmed. In addition, there was a reduction in the expression of β-catenin, phospho-β-catenin, C-myc, and survivin as a result of curcumin [79]. The effect of curcumin on selected GC carcinogenesis processes is presented below in Table 3.

### 4.2. Clinical Trials Based on Curcumin in Gastric Cancer Therapy

Global specialists are trying to find an appropriate way, i.e., clinical trials or experimental therapies, which will enable a significant improvement in the quality of diagnostics and treatment of patients with gastric cancer. Patient participation in a clinical trial allows for premature access to therapy, which would be widely available in a few years. However, there are appropriate criteria for patient participation, in accordance with the adopted protocol and specific rules. Among the most important goals of clinical trials are the following: the desire to find a cure for a given disease and to improve the quality of life. On the other hand, conducting specific clinical trials provides an answer regarding whether a new approach to treating a given disease is more effective and better tolerated than the treatment methods currently available on the market.

There are three clinical trials registered on the clinicaltrials.gov website in 2024 (presented in Table 4), that evaluate the effectiveness of curcumin in the treatment of gastric cancer. One of them is not yet recruiting, another is already recruiting, and the third is active but not recruiting.

In the case of the NCT05856500 test, these will be controlled tests, which involve the oral administration of curcumin together with creatine. The experiment aims to see if such a combination can reduce inflammation, improve disruption in normal nutrient metabolism, and significantly improve the prognosis of patients. In the NCT02782949 study, the main goal is to compare the concentration of interleukin 1β (IL-1beta) in the gastric mucosa of patients using curcumin over a period of 6 months with placebo patients. The results are already available, but according to the National Library of Medicine, they appear to be inconsistent.

## 5. Quercetin

**Figure 4:**
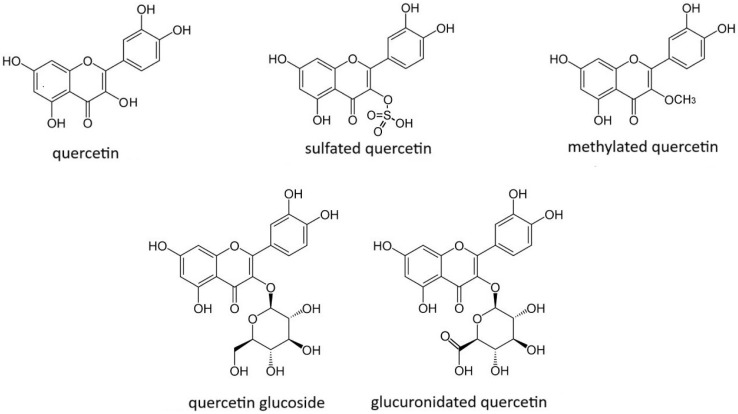
Forms of quercetin metabolites.

Quercetin (3,3′,4′,4,7-pentahydroxyflavone) (forms presented in Figure 4), which chemically belongs to flavonoids, or more precisely, flavonols, is an organic polycyclic aromatic compound of plant origin [80]. It occurs in a yellow-colored crystalline form, and is most often found in the form of glycosides, although it is also often found in free form. It has a flat molecular structure, so it can more easily penetrate deep into the lipid bilayer protecting lipids from peroxidation. It is obtained during crystallization from plant extracts, with it being practically insoluble in water. It exhibits a lipophilic character and considerable bioavailability from the gastrointestinal tract. In dissolved form, it is found in red wine and green tea, while in free form, it is found in hawthorn flowers and chestnut trees, among others [81]. It can also be found in such plants as dark grapes, apples, elderberries, rosehips, cherries, black currants, strawberries, cranberries, chokeberries, blueberries, and raspberries [82].

The health-promoting functions of quercetin include lowering the LDL fraction more effectively [83], exhibiting strong antioxidant activity due to its ability to neutralize the free radicals present both in food and in human body cells, its ability to chelate metal ions, inhibit the activity of oxidases, and lipid peroxidation [84]. Quercetin consumption is also associated with a reduced risk of cardiovascular disease. Studies have shown that quercetin is the most effective inhibitor of platelet aggregation, which has applications in the treatment of thromboembolic vascular disease [85]. Quercetin administered with calcium salts, as well as its derivatives, including rutin, exhibits anti-allergic properties due to the inhibition of the production and release of histamine and other mediators of the allergic reaction [86]. In addition, quercetin exhibits antimicrobial activity. In vitro studies show that it inhibits the growth of *Helicobacter pylori* bacteria, playing an important role in the prevention and treatment of peptic ulcer disease [87]. Quercetin also has antiviral activity. This compound, along with myricetin, inhibits HIV integrase. Polyphenolic compounds restrict the entry of HIV-1 virus particles into CD4 lymphocytes and exhibit viral reverse transcriptase antagonist activity. Quercetin’s antiviral activity involves binding to the virus’ coat proteins, leading to their inactivation and DNA destabilization. It also reduces the activity of herpes virus (HSV), rabies, influenza, and polio viruses [88]. Quercetin also exhibits protective effects on healthy cells in the body, blocking the cell cycle, initiating apoptosis by modifying the expression of signaling proteins and receptors, as well as re-framing intracellular pathways [89]. In addition, it has been shown that flavonoids, mainly quercetin, can exhibit positive effects on fat mass reduction by inducing the apoptosis of adipocytes, inhibiting their formation, or increasing their lipolysis [90].

### Quercetin and Gastric Cancer

A study was conducted in which the effects of different concentrations of quercetin on GCs were examined. CCK-8 analysis showed that AGS cell growth was significantly inhibited after quercetin treatment, and the degree of inhibition increased with increasing quercetin concentrations. Next, the effects of different concentrations of quercetin on the expression levels of protein markers of pyroptosis and the apoptosis of AGS cells were examined. As expected, the expression levels of pyroptosis markers (GSDMD, GSDME, Cleaved CASP1, NLRP3) were significantly elevated in quercetin-treated AGS cells relative to AGS cells without quercetin intervention, and the expression levels increased with increasing drug concentration, suggesting that quercetin activates essential pyroptosis genes, such as GSDMD, to promote the scavenging pathway in GC cells, and that this effect is dependent on drug concentration. In addition, quercetin increased the expression of CASP3 and PARP in AGS cells, suggesting that AGS also induced apoptosis [91].

In a study conducted on MKN-45 cells, the effect of different concentrations of quercetin 0, 20, 40, and 60 μmoL/L for 24 h was studied. The optimal concentration turned out to be 40 μmoL/L. Pro-apoptotic and proliferative effects were confirmed by significantly reducing the expression of TP53, TIMP1, and MYC [92].

Another study used MKN-45 GC cells that were incubated at a concentration of 25 μM of the active component of licorice and quercetin. It has been shown that this substance significantly blocks proliferation but also inhibits the cell cycle and stimulates apoptosis in cancer cells. As a result, there was an increase in Cyt-C and a decrease in Bcl-2 expression, as well as mitochondrial membrane potential, and, consequently, a mitochondrial defect. In addition, quercetin was shown to block the ERK-related signaling pathway. In addition, an in vivo study was performed using a mouse model that administered the test substance at a concentration of 20 mg/kg daily for 15 days. In this case, quercetin minimized the tumor size, along with a decrease in the expression of BCL-2 and Ki-67 in the tumor tissue. An increase in caspase 3 and BAX levels was also observed [93].

Treatment failure due to multidrug resistance (MDR) is a key obstacle during chemotherapy. Multidrug resistance is generally correlated with the upregulation of adenosine triphosphate (ATP) binding cassette (ABC) transport proteins. In addition, the abnormal activation of the phosphoinositide kinase 3 (PI3K)/protein kinase B (Akt) pathway may counteract chemotherapeutic induction. The identification of safe and effective MDR-reversing compounds is essential for gastric cancer therapy. To this end, we investigated the role of quercetin in mediating the expression and activity of the osmotic glycoprotein (P-gp) as an ABC transporter in the PI3K/Akt/P-gp cascade in the oxaliplatin-resistant (OxR) gastric cancer cell line KATOIII/OxR. The results showed that quercetin increased the cytotoxicity of OxR and decreased the expression and activity of P-gp, and reversed MDR by increasing the cytotoxicity of intracellular OxR in KATOIII/OxR cells and affected the rate of apoptosis in KATOIII/OxR cells. Quercetin induced a dramatic reduction in KATOIII/OxR cell survival and may reverse OxR resistance by reducing P-gp expression and activity. These data suggest that the exposure of KATOIII/OxR cells in a dose-dependent manner to quercetin may circumvent MDR by improving the intracellular accumulation of OxR, leading to the theory that quercetin may provide a new treatment strategy and improve the survival of patients after chemotherapy for gastric cancer [94].

The miR-145/quercetin combination therapy study examined the effect of quercetin on autophagy. The results showed that miR-143 sensitized AGS and MKN28 to quercetin in a synergistic manner, and the combination therapy could improve the therapeutic response of quercetin by inhibiting autophagy. We also found that the expression of autophagy marker proteins, LC3I/II, and the miR-143 target, GABARAPL1, increased when cells were treated with quercetin, while, in contrast, it decreased in the combination therapy group and after the inhibition of cell autophagy by miR-143 treatment alone. As a result, these data indicate that miR-143 can enhance quercetin chemosensitivity by inhibiting autophagy in GC cells [95]. In subsequent studies, quercetin treatment resulted in the formation of numerous microscopic vacuoles, with autophagic vacuoles being the main feature of autophagy. Compared to the control group, these observations suggest that quercetin’s effect is related to autophagy. Further examination by electron microscopy revealed a decrease in mitochondrial size and an increase in membrane density, which indicate ferroptosis, a significant phenomenon. Meanwhile, an immunofluorescence assay was performed to detect changes in TfR1 and ATG5. As revealed by the results after quercetin treatment, TfR1 expression was reduced, while ATG5 increased. To investigate the mechanisms of quercetin’s effect on AGS and MKN45 cell viability, siATG5 was used to detect the effect of quercetin on ferroptosis and autophagy. The results of the RT-PCR assay showed the successful formation of siATG5 in AGS and MKN45 cells. CCK-8 assay and cell colony formation assay were performed to detect cell viability. As the results showed, cell viability was significantly reduced after quercetin treatment compared with the control group. To further investigate the effect of quercetin on ROS levels, flow cytometry was performed. The results presented here showed that ROS levels were significantly increased after quercetin treatment compared to the control group. In this study, the effect of quercetin on the expression of ATG5, LC3B, and Beclin-1 was also determined, and for this purpose, immunofluorescence assay was performed. As it was shown, the levels of ATG5, LC3B, and Beclin-1 were significantly increased after quercetin treatment. The next aim was to determine the effect of quercetin on the expression of TfR1, GPX4, and SLC7A11, and for this purpose, Western blot assay was performed. The results showed that the levels of TfR1, GPX4, and SLC7A11 were significantly decreased after quercetin treatment. This study was also performed on BALB/c mice that were injected subcutaneously with AGS cells in the left armpit area. The administration of quercetin to xenograft mice led to reductions in TfR1, SLC7A11, and GPX4 levels [96]. The effect of quercetin on selected GC carcinogenesis processes is presented below in Table 5.

Most of the gastric cancer patients have metastasis of cancer cells at the time of diagnosis. Cisplatin can slow down the development of gastric cancer, but drug resistance will develop after a long time of chemotherapy. Previous studies have shown that quercetin improves the resistance to chemotherapy drugs. Therefore, this study aims to investigate the role of quercetin in gastric cancer. The drug-resistant cell line SGC-7901 was cultured and treated. The MTT assay evaluated the cell proliferation, cell survival rate, IC50 value, and sensitivity along with the analysis of cell apoptosis, proliferation by colony formation assay, gene expression by qRT-PCR (real-time reverse transcription), and detection of FOXD3 (Forkhead box D3) levels by Western blot. A drug-resistant cell model of gastric cancer was successfully established, and quercetin inhibited the cell survival to a certain extent and improved its chemosensitivity. The proapoptotic effect of quercetin on cisplatin chemotherapy resistance in gastric cancer is associated with increased FOXD3 levels. Quercetin can directly regulate the expression of FOXD3, which is the effect of activation. In conclusion, quercetin has a strong anticancer effect. It can inhibit the activity of gastric cancer cells and accelerate apoptosis by activating the FOXD3 signaling pathway [97]. The effect of quercetin on the level of VEGF-R2 and VEGF-A in tumor tissues and blood during the period of irinotecan treatment was studied. It was found that the level of VEGF-R2 in tumor tissues in the quercetin-treated groups was lower than in the vehicle control group. Furthermore, when quercetin was administered during the period of irinotecan treatment, the level of VEGF-A in tumor tissues was significantly reduced compared with the control group [98].

Several in vivo studies have been conducted in male BALB/c mice that were injected with AGS tumor cells in the right flank area. Some mice were then injected with 20 mg/kg of quercetin, while others were injected with quercetin and irinotecan (topoisomerase I inhibitor) for 3 days during the week. The experiment lasted 4 weeks. In the case of quercetin alone and a combination of drugs, a decrease in VEGF-A levels in diseased tissues was observed compared to the control group. However, the use of both drugs has been shown to be more satisfactory. Research also included COX-2, which is an important factor in promoting metastasis. It has been shown that the use of a combination of drugs significantly reduces the expression of COX-2 in tumor tissues [98].

In a study conducted on NOD/SCID xenotransplant mice, they were additionally injected with GC EBV (+) or EBV (−) cells—SNU719 or MKN74. Quercetin (30 mg/kg) was then administered orally over a period of 2 weeks. In the course of the 3-week experiment, it was noted that from day 5 onwards, the growth inhibition of human GC positive EBV (SNU719) began, and a sharp increase was observed from day 9. In EBV-negative cancer (MKN74), quercetin manifested itself as a delayed mechanism of disease inhibition, which was noticed only on the 13th day of the experiment. Studies have shown that quercetin has potential in the treatment of human GC correlated with EBV [99].

**Figure 5:**
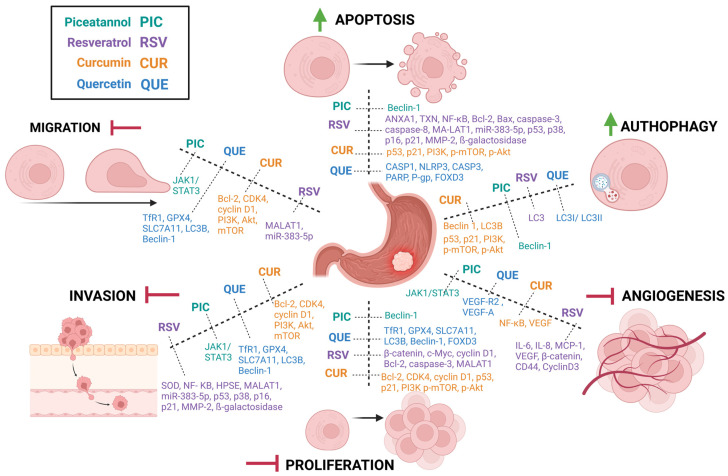
Summary of how natural products can influence the process of carcinogenesis in gastric cancer, with the objectives of molecular research highlighted. Created in BioRender. Poniewierska-Baran, A. (2024) https://BioRender.com/y52c684 (accessed on 30 October 2024).

The effect of how natural products can influence the process of carcinogenesis in gastric cancer, with the objectives of molecular research highlighted is presented below in Figure 5.

## 6. Conclusions

Unhealthy dietary habits, which are a consequence of functioning in a developing society, are one of the most important causes of the gastric carcinogenesis process, as well as other parts of the digestive system. It has been proven that regular daily consumption of fruits and vegetables protects against diseases and the development of cancers, which has been confirmed by the results of many case-control studies presented in this paper. Eating hot, salted, fried meals, but also pickled or smoked foods, as well as nitrogenous substances and aromatic hydrocarbons are important factors, the consumption of which increases the risk of gastric cancer. As was shown by Maddineni et al. [100], the introduction of vegetables and fruits to the diet can reduce the risk of developing GC disease by up to 66–75%. These are results that cannot be ignored.

Naturally occurring polyphenolic compounds, such as resveratrol, piceatannol, curcumin, and quercetin, have garnered significant attention due to their diverse biological activities and potential roles in cancer prevention and treatment. Interestingly, despite the proven impact of these substances on GC cell lines, studies in GC animal models, or groups of patients, only curcumin has been introduced as an important therapeutic target in clinical trials for gastric cancer. This is probably due to the low bioavailability of these substances, so learning how to deliver and administer a well-bioavailable form of polyphenolic compounds will probably be one of the key issues to consider in this topic. It should be remembered that these substances have many health benefits for the entire body, like the cardiovascular system and the nervous system, they improve metabolism and help fight obesity, and they also affect our immune system by reducing inflammation, which, if prolonged, can ultimately lead to the start of the cancer process and its metastasis.

The small number of in vivo studies (especially involving GC patients) as well as clinical trials using natural products like resveratrol, curcumin, piceatannol, and quercetin indicates a big scientific gap that needs to be filled, especially considering the satisfactory results of in vitro studies, which confirm the significant effect of these substances on the process of GC cell destruction or inhibition of their progression. The main problem is poor bioavailability or activity, which of course can be solved by modifying their structure or form or the dosing way. The pro-health effect of resveratrol, curcumin, piceatannol, and quercetin on patients with GC is beyond dispute, so now the next step should be extensive research on modifying their bioavailability and improving their absorption.

The contribution of natural compounds with low drug resistance and few side effects, as well as the proposed drug combination therapeutics, can bring new developments in the treatment of GC [101]. Our work explored the connection between four representative natural compounds and gastric cancer progression, with a focus on targeted therapies via important signaling pathways and molecular targets in GC. We hope that this will point the direction of novel therapeutic options for GC therapy.

It is necessary to provide more scientific data, widely observed for centuries, that a diet including polyphenolic compounds, such as resveratrol, piceatannol, curcumin, and quercetin, has general health-promoting effects, including anticancer effects. Thanks to advanced biomedical research and the development of techniques for assessing cell signaling and gene and protein expression, we are able to indicate the therapeutic targets that these compounds affect, and learn the mechanism of their action as well as the precise impact on cancer cells’ biology.

## Figures and Tables

**Table 1:** The influence of resveratrol on selected process of GC carcinogenesis.

Process of Carcinogenesis	Model	Dose	Influence on GC	Molecular Target of Interest	References
Apoptosis	HGC-27, AGS EPG85-257 (RDB), EPG85-257 (RNOV), SGC-7901	100 μg/mL 10,20,30,40,50,200, 400 µM	↑	↓↑ANXA1,↓TXN, ↓NF-κB, ↓Bcl-2, Bax, ↑caspase-3, ↑caspase-8, ↓MALAT1/miR-383-5p, ↑p53, ↑p38, ↑p16, ↑p21, ↑MMP-2, ↑ß-galactosidase	[7,22,24,26,27,28]
Autophagy	HGC-27	50 µM	↓	↓LC3	[29]
Angiogenesis	GC–MSCs, HGC-27, AGS	20 µM	↓	↓IL-6, ↓IL-8, ↓MCP-1, ↓VEGF, ↓β-catenin, ↓CD44, ↓CyclinD3	[30]
Proliferation	SGC7901, GES-1, MGC803, AGS	25, 50, 75, 100, 200 (μM)	↓	↓miR-155-5p, ↓claudin-1, ↓Wnt/β-catenin, ↓c-Myc, ↓cyclin D1, ↓Bcl-2, ↑caspase-3, ↓MALAT1/miR-383-5p	[6,23,26]
Invasion	AGS, MKN45, BGC823	25–200 μM	↓	↑SOD, ↓NF-KB, ↓HPSE, ↓MALAT1/ miR-383-5p, ↑p53, ↑p38, ↑p16, ↑p21, ↑MMP-2, ↑ß-galactosidase	[5,25,26]
Migration	BGC823	200 µM	↓	↓MALAT1/miR-383-5p	[25,26]
ROS	AGS	100 µM	↑	-	[28]

Abbreviations: ANXA1, annexin A1; TXN, thioredoxin; NF-κB, nuclear factor kappa B; Bcl-2, B-cell lymphoma 2; Bax, bcl-2-like protein 4; MALAT, metastasis associated lung adenocarcinoma transcript 1; MMP-2, matrix metalloproteinase-2; LC3, microtubule-associated proteins 1A/1B light chain 3, IL-6, interleukin 6; IL-8, interleukin 8; MCP-1, monocyte chemoattractant protein 1; VEGF, vascular endothelial growth factor; SOD, superoxide dismutase; HPSE, heparanase; c-Myc, proto-oncogene; ROS, reactive oxygen species; ↑ upregulation; ↓ downregulation.

**Table 2:** The influence of piceatannol on selected process of GC carcinogenesis.

Process of Carcinogenesis	Model	Dose	Influence on GC	Molecular Target of Interest	References
Apoptosis	SGC7901, BGC823, MKN28, MGC803, HGC27, and AGS	5 μmol L^−1^	↑	↑Beclin-1, ↑Cacspase-9-Caspase-3, ↑PARP, ↓Bcl-2,	[8]
Autophagy	SGC7901, BGC823, MKN28, MGC803, HGC27, and AGS	5 μmol L^−1^	↑	↑Beclin-1, ↑LC3, ↓SQSTM1/p62	[8]
Angiogenesis	SGC-7901	10, 20 µM	↓	↓JAK1/STAT3	[67]
Proliferation	SGC7901, BGC823, MKN28, MGC803, HGC27, and AGS	5 μmol L^−1^	↓	↑Beclin-1, ↑LC3B, ↑PARP	[8]
Invasion	SGC-7901	10, 20 µM	↓	↓JAK1/STAT3	[67]
Migration	SGC-7901	10, 20 µM	↓	↓JAK1/STAT3	[67]

Abbreviations: JAK1, Janus kinase 1; STAT3, signal transducer and activator of transcription 3; SQSTM1/p62, selective autophagic marker; LC3, microtubule-associated proteins; PARP, poly (ADP-ribose) polymerase; Bcl-2, B-cell lymphoma 2; ↑ upregulation; ↓ downregulation.

**Table 3:** The influence of curcumin on selected process of GC carcinogenesis.

Process of Carcinogenesis	Model	Dose	Influence on GC	Molecular Target of Interest	References
Apoptosis	SGC-7901, hGCC, BGC-823	10,20,40 μM	↑	↑p53, ↑p21, ↓PI3K, ↓mTOR, ↓Akt β-catenin	[73,79]
Autophagy	SGC-7901, AGS, BGC-823, HGC-27	10,20,40 μM	↑	↑p53, ↑p21, ↓PI3K, ↓mTOR, ↓Akt, ↑ATG5, ↑ATG7, ↑Beclin 1, ↑LC3B	[73,75]
Angiogenesis	GC-MSC	30 μM	↓	↓NF-κB, ↓VEGF	[77]
Proliferation	SGC-7901, hGCC, BGC-823,	10,20,40 μM	↓	↓Bcl-2, ↓CDK4, ↓cyclin D1, ↑p53, ↑p21, ↓PI3K, ↓mTOR, ↓Akt	[71,73]
Invasion	SGC-7901, AGS HGC-37	20, 30, 50, 75, 100 μM	↓	↓Bcl-2, ↓CDK4, ↓cyclin D1, ↓circ_0056618, ↓PI3K, ↓Akt, ↓ mTOR	[71,72,74,76]
Migration	SGC-790, HGC-37, AGS, hGCC	20, 30, 50, 75, 100 μM	↓	↓Bcl-2, ↓CDK4, ↓cyclin D1, ↓circ_0056618, ↓PI3K, ↓Akt, ↓mTOR	[71,72,74,76]
ROS	hGCC	20 μM	↑	-	[76]

Abbreviations: Bcl-2, B-cell lymphoma 2; CDK4, cyclin-dependent kinase 4; PI3K, phosphoinositide 3-kinases; Akt, protein kinase B; mTOR, mammalian target of rapamycin; NF-κB, nuclear factor kappa B; VEGF, vascular endothelial growth factor; LC3B, microtubule-associated proteins 1A/1B light chain 3B; mTOR, mammalian target of rapamycin; Akt, serine/threonine kinase; ATG5, autophagy-related 5; ATG7, autophagy-related 7; ↑ upregulation; ↓ downregulation.

**Table 4:** Clinical studies using curcumin in gastric cancer (GC) treatment.

Trial Number	Conditions	Status/Phase	Age (Years)	Locations
NCT05856500	Stage IIIA Gastric Cancer, Stage IIIB Gastric Cancer, Stage IV Gastric Cancer	not yet recruiting	18–80	location not provided
NCT04871412	Gastric Cancer	recruiting	≥18	Canada
NCT02782949	Chronic Atrophic Gastritis	active, not recruiting	≥21	Honduras, Puerto Rico

**Table 5:** The influence of quercetin on selected process of GC carcinogenesis.

Process of Carcinogenesis	Model	Dose	Influence on GC	Molecular Target of Interest	References
Apoptosis	AGS, KATOIII/OxR SGC-7901	20,40,80 μM	↑	↑GSDMD, ↑GSDME, ↑CASP1, ↑NLRP3, ↑CASP3, ↑PARP, ↑P-gp, ↑FOXD3, ↓TP53, ↓TIMP1, ↓MYC, ↑Cyt-C, ↓Bcl-2	[91,92,93,94,97]
Autophagy	AGS, MKN28	40, 150 µM	↑	↑LC3I/LC3II	[95]
Angiogenesis	xenograft model nude mice/AGS	20 mg/kg	↓	↓VEGF-R2, ↓VEGF-A	[98]
Proliferation	AGS, MKN45 SGC-7901	20, 40, 80, 160, 320, 640 μM	↓	↓TfR1, ↓GPX4, ↓SLC7A11, ↑LC3B, ↑Beclin-1, ↑FOXD3, ↓TP53, ↓TIMP1, ↓MYC	[92,96,97]
Invasion	AGS, MKN45	20, 40, 80, 160, 320, 640 μM	↓	↓TfR1, ↓GPX4, ↓SLC7A11, ↑LC3B, ↑Beclin-1	[96]
Migration	AGS, MKN45	20, 40, 80, 160, 320, 640 μM	↓	↓TfR1, ↓GPX4, ↓SLC7A11, ↑LC3B, ↑Beclin-1	[96]
ROS	AGS, MKN45	20, 40, 80, 160, 320, 640 μM	↑	-	[97]

Abbreviations: GSDMD, gasdermin D; GSDME, gasdermin E; CASP1, caspase-1; NLRP3, NLR family pyrin domain containing 3; CASP3, caspase-3; PARP, poly (ADP-ribose) polymerase; P-gp, p-glycoprotein 1; FOXD3, forkhead box D3; LC3I, microtubule-associated proteins 1A/1B light chain 3 I; LC3II, microtubule-associated proteins 1A/1B light chain 3 II; VEGF-R2, vascular endothelial growth factor receptor2; VEGF-A, vascular endothelial growth factor A; TfR1, transferrin receptor 1; GPX4, glutathione peroxidase 4; SLC7A11, solute carrier family 7, member 11; ↑ upregulation; ↓ downregulation.

## Data Availability

Not applicable.
